# Persistent Luminescent Nanoparticle-Loaded Filaments for Identification of Fabrics in the Visible and Infrared

**DOI:** 10.3390/nano14171414

**Published:** 2024-08-29

**Authors:** Brian G. Yust, Abdur Rahaman Sk, Antonios Kontsos, Brian George

**Affiliations:** 1 College of Humanities & Sciences, Thomas Jefferson University, Philadelphia, PA 19144, USA; 2 Research and Development, Lear Corporation, 1 Penn-Dye St, Pine Grove, PA 17963, USA; abdurrahaman45@gmail.com; 3 College of Engineering, Rowan University, Glassboro, NJ 08028, USA; kontsos@rowan.edu; 4 School of Design & Engineering, Thomas Jefferson University, Philadelphia, PA 19144, USA; brian.george@jefferson.edu

**Keywords:** nanoparticles, nanomaterials, nanocomposites, persistent luminescence, afterglow, near infrared, filaments, fabrics, textiles

## Abstract

Persistent luminescent materials are those which can store an amount of energy locally and release it slowly in the form of light. In this work, persistent luminescent nanoparticles (PLNPs) were synthesized and incorporated into polypropylene (PP) filaments at various loading percentages. We investigated the optical properties of both the as-prepared PLNPs and the PLNP-loaded filaments, focusing on any changes resulting from the integration into the filaments. Specifically, visible and near-infrared spectroscopy were used to analyze the emission, excitation, and persistent luminescence of the PLNPs and PLNP-loaded filaments. The tensile properties of the extruded filaments were also investigated through breaking tenacity, elongation at break, Young’s modulus, and secant modulus. All PLNP-loaded filaments were shown to exhibit persistent luminescence when exposed to ultraviolet light. While there were no significant changes in the elongation at break or Young’s modulus for the loading percentages tested, there was a slight increase in breaking tenacity and a decrease in the secant modulus. Finally, the filaments were shown to maintain their optical properties and persistent luminescence even after abrasion testing used to simulate the normal wear and tear that fabric experiences during use. These results show that PLNPs can be successfully incorporated into filaments which can be used in fabrics and will maintain the persistent luminescent properties.

## 1. Introduction

Nanoparticles (NPs) which include both fluorescent and optically active types are increasingly being used in everyday products due to recent improvements in production efficiency and cost-effectiveness [1,2]. Rare earth elements, also known as the lanthanides, are widely employed for their unique optical characteristics across various fields, including optical communications [3,4], energy storage [5,6], digital displays [7,8,9], and biomedical imaging [10,11]. While silver NPs have been effectively implemented in textiles and clothing for their antimicrobial attributes [12,13,14,15,16,17], there has been limited exploration into rare-earth-doped NPs as they are integrated into textiles and fabrics, which could be of significant interest to multiple industries. In this study, we focus on an extrusion method of integration of rare-earth-based persistent luminescent nanoparticles (PLNPs) into filaments to be used in textiles. Once incorporated, the PLNP-loaded filaments’ optical properties allow for visible or near-infrared identification. The PLNPs were synthesized using a two-step hydrothermal synthesis followed by high-temperature sintering in an atmospheric environment. During the final sintering step at sufficiently high temperatures, additional oxygen vacancies are generated within the host nanoparticle crystal structure. This enables energy storage and gradual release, observed as persistent luminescence. Visible and near-infrared spectroscopy were used to measure emission under ultraviolet excitation while the PLNP loading percentage was varied to optimize emission intensity without compromising filament strength.

Persistent luminescence is the phenomenon where a material can absorb and store energy locally and release it slowly in the form of light. A few of the most commonly known applications include glow-in-the-dark stickers and toys as well as Indiglo™ watches. A wave of research and application came in the mid-1990s with the introduction of the efficient Eu^2+^ and Dy^3+^ co-doped aluminates SrAl_2_O_4_:Eu^2+^, Dy^3+^ [18,19] and CaAl_2_O_4_:Eu^2+^, Dy^3+^ [20]. The persistent luminescence duration, commonly referred to as afterglow, was extended to more than 24 h and allowed for the use of natural sunlight as an excitation source. This extended duration of luminescence and the substitution of an external excitation source with sunlight was useful for practical applications [18,19,21]. Rare earth materials are widely used for optical applications due to their unique spectral qualities. Rare-earth-based NPs which exhibit persistent luminescence often require high-temperature sintering to create vacancies or defects in the NP crystal structure which act to store absorbed energy and release it as luminescence over a long period of time. The resulting emission may last on the order of seconds to hours and may be described as phosphorescence (short lasting or long lasting) or afterglow.

While the exact physical mechanisms which lead to persistent luminescence are still being actively researched, it is generally accepted that crystal defects and vacancies in the host crystal act as traps for charge carriers in a material [21,22,23]. When the charge carriers in a material are excited, either by absorption of light or some other means, some of the charge carriers will become trapped at these vacancy sites. Gradually, these trapped carriers are released, and their energy can be transferred to luminescent sites such as the rare-earth dopants also present in the crystal which, in turn, release the energy as light [22,24,25]. The most successful models for explaining this behavior describe the energy levels of the light-emitting dopants and energy traps as being located within the bandgap of the host material [25,26,27]. There are numerous rare-earth-doped materials which have successfully been used as persistent luminescent materials including aluminates, silicates, glasses, and sulfides [22]. Specifically for Eu-doped materials such as those used here, an electron trapping and de-trapping model is widely used to explain the phenomenon [19,20,21,22,23], in which an electron from the Eu site is excited by an incident photon to the conduction band and then migrates to a long-lived energy state slight lower than the conduction band, the “trap”. This trap may be a crystal defect, such as an oxygen vacancy in LaAlO_3_, or a co-dopant with an appropriate energy level. Once the electron is trapped, it will stay there for some amount of time and may spontaneously be thermally excited back into the conduction band and transfer to a luminescence site, an allowed excited Eu state, which then de-excites and emits.

Recently, nanomaterials which retain this persistent luminescent property have become more sought after due to the numerous applications of optically active nanomaterials ranging from biomedical imaging to textiles [19,24,27]. Optically active NPs such as rare-earth-doped metal oxides have been successfully incorporated into various matrices including polyurethane [28], polymethylmethacrylate [29], polylactic acid [30], polyethylene glycol [30,31], and cellulose [32,33]. These luminescent NPs can also be incorporated into various composite materials such as films, filaments, fibers, or molded pieces by various methods. In the typical luminescence process, NPs emit certain colors of light while being exposed to an excitation source such as visible light, UV radiation, electron beam, plasma beam, X-rays, or even γ rays. This excitation energy is absorbed and re-emitted at a different wavelength due to small, non-radiative energy losses. In persistent luminescence, the excitation energy is absorbed and stored at an energy trap site such as crystal defects in NPs [19,24,30,31]. The crystal defects can be intrinsic or intentionally created by sintering at high temperature. The excitation energy is stored at these defect sites for some period of time and then slowly released to a luminescent center where it is converted to light and emitted. 

Previous work has shown that luminescent NPs can be incorporated into polymers and fibers. For example, SrAl_2_O_4_: Eu^2+^, Dy^3+^, Sr_2_ZnSi_2_O_7_: Eu^2+^, Dy^3+^ and Y_2_O_2_S: Eu^3+^, Mg^2+^, Ti^4+^ were synthesized and incorporated into luminous fibers which also exhibited persistent luminescence in the visible spectrum [34]. Similarly, Kulpinski et al. used cerium fluoride particles and eight percent by weight cellulose solution in N-methylmorpholine-N-oxide (NMMO) to obtain luminescent fibers by wet spinning [35]. Yu et al. demonstrated that Eu-doped yttria NP-loaded nanofibers could be prepared by electrospinning [36]. After the nanofibers were dried, calcining them at a high temperature yielded better luminous properties. Ge et al. examined microstructure, composition, and afterglow properties of luminescent fibers by using polyethylene terephthalate (PET) as a matrix through a melt spinning process [37]. Another study mixed SrAl_2_O_4_:Eu^2+^, Dy^3+^ NPs with PET and then used a twin screw extruder to produce luminescent fibers [38]. Chen et al. experimented with the preparation of SrAl_2_O_4_ doped with Eu and Dy by using different concentrations of luminescent NPs ranging from 0.1% to 1.4%, to analyze the effect on the luminescent effect of the fiber melt spinning process when incorporating into polyamide-6, which also exhibited persistent luminescence in the visible spectrum [39]. Lozano et al. incorporated Zn_2_GeO_4_ NPs doped with Mn^2+^ or Cr^3+^ into polyvinyl alcohol (PVA) fibers which showed green and red persistent luminescence, respectively [40].

Thus far, nearly all fundamental and applied research on luminescent or persistent luminescent textile materials has focused on emission in the visible light region, but there has not been much exploration of either luminescence or persistent luminescence in the non-visible regions such as the infrared. Beyond the novelty of incorporating materials with persistent luminescence in the infrared into fabrics, there are unexplored real-world applications for low-light identification of individuals. Specifically, tactical missions and search-and-rescue operations could benefit greatly from their use, providing an ability to identify and locate individuals at a distance in low-light situations without the need for an external light source to illuminate the individual like a spotlight or laser. This work addresses this gap in knowledge about persistent luminescence in filaments and textiles at wavelengths at or near the infrared. To achieve this, LaAlO_3_ persistent luminescent nanoparticles (PLNPs) doped with Eu and Zn were chosen as the host–dopant combination. Zn was included as a co-dopant in the samples tested here to extend the persistent luminescence lifetimes. It has been found in similar systems that the Zn can act as an electron trap due to energy levels slightly below those of the conduction bands for some materials [19,20,21,22]. Once the synthesis method was optimized to yield the brightest and longest persistent luminescence, the PLNPs were incorporated into polypropylene (PP) filaments using an extrusion method while maintaining their optical properties. These persistent luminescent filaments could then be incorporated into textiles or fabrics.

## 2. Materials and Methods

### 2.1. Nanoparticle Synthesis

Persistent Luminescent Nanoparticles (PLNPs) composed of LaAlO_3_:Eu, Zn were synthesized via a wet chemical method adapted from Lin et al. [41]. Briefly, stoichiometric amounts of precursors (LaNO_3_, AlNO_3_) and dopants (EuCl_2_, ZnNO_3_) were mixed in deionized water and heated to 80 °C on a hot plate while under vigorous stirring. Ammonium hydroxide was added dropwise to initiate nucleation and growth of hydroxide nanocrystals while the solution was held at temperature for 30 min. This solution was then placed in a steel autoclave (Baoshishan 100 mL polytetrafluoroethylene (PTFE)-lined hydrothermal synthesis reactor) and heated to 120 °C in an oven (Thermolyne L-50-4 model furnace) for 24 h. After cooling to room temperature, the resulting precipitate was centrifuged (Thermo Fisher Scientific model Sorvall Lynx 4000), sonicated, and reconstituted in solution with deionized water three times. Finally, PLNP samples were dried under ambient atmosphere in a low-temperature oven heated to 65 °C. A second heat treatment of the PLNP powders was carried out in a Lindberg/Blue M, TF55035A-1 tube furnace at 1000 °C for 2 h.

### 2.2. Filament Extrusion

Filaments containing PLNPs were produced using a Haake Mini CTW twin screw extruder at 190 °C and 100 rpm. Filaments were prepared with PLNP loading of 0.5%, 1%, and 5% by weight. The PLNP powder was mixed directly with polypropylene beads into the hopper on the extruder, followed by the extrusion process. The formation ability of the luminescent filaments deteriorated when the PLNP loading was greater than 5% because the melt became too viscous. At 10% PLNP loading, the melt did not come out of the spinneret, resulting in the extruder becoming jammed. Filaments remained in an un-drawn state after extrusion.

### 2.3. Spectroscopy

Absorption and emission spectroscopy in the UV, visible, and near-infrared ranges were conducted on PLNP powder and filament samples. Emission spectra, excitation spectra, and persistent luminescence decay curves of PLNP powder and filament samples were obtained using a Horiba Fluoromax 4 (Edison, NJ, USA) or Ocean Optics Flame VIS-NIR spectrometer (Orlando, FL, USA). A 450 nm long-pass filter (Thorlabs FEL450 (Newton, NJ, USA)) was used to filter out lines from the excitation lamp. Persistent luminescence decay curves were normalized and fitted using a double-exponential decay function using Origin Lab (Northampton, MA, USA).

### 2.4. Filament Testing

Young’s modulus was calculated by taking the slope of the initial section of the stress–strain graph. Logger Pro 3.15 is a software tool for statistical analysis. To calculate the Young’s modulus, Logger Pro 3.15 software was used. By plotting stress–strain data from an Instron 6800 universal testing system, linear fit and initial slope of the stress–strain graph were obtained. Young’s modulus was calculated by taking a linear fit of the initial slope of the stress–strain in kilogram-force/square mm, then converted to MPa. The secant modulus was calculated using a linear fit of the stress–strain graph between the origin and either 2% or 4% strain, as denoted. Tensile testing of single end filaments was also carried out on the Instron 6800 with 62 mm gauge length at 370 mm/minute test rate. The samples were conditioned in the lab at 65% RH and 20 °C for four hours prior to evaluation.

### 2.5. Imaging

Scanning electron microscopy was performed using a Hitachi FlexSEM 1000 (Hitachi High-Tech, Tokyo, Japan). Nanoparticle powder samples were mixed in a solvent (water or ethanol) and dried on a metal substrate or mesh grid. Filament samples were attached to the sample puck using conducting tape, and the stage was rotated to achieve the desired viewing angle. A Skyscan 1172 Micro-CT scanner (Bruker, Allentown, PA, USA) was also used to visualize the 3D distribution of the PLNPs throughout the filament samples with an 8.18-micron resolution.

## 3. Results

### 3.1. Persistent Luminescent Nanoparticles (PLNPs)

**Figure 1:**
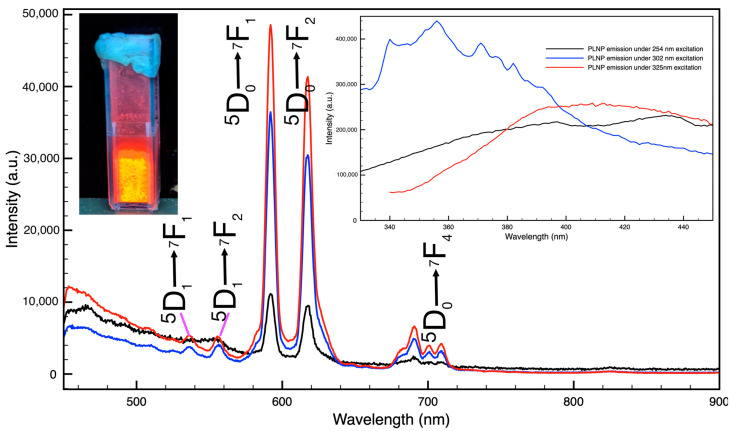
Emission spectra of LaAlO_3_: Eu, Zn PLNPs under 254 nm, 302 nm, and 325 nm. Left inset shows a visible image of the PLNPs in a cuvette under 302 nm excitation. Right inset shows emission in the 330–450 nm spectral window taken without the long-pass filter.

**Figure 2:**
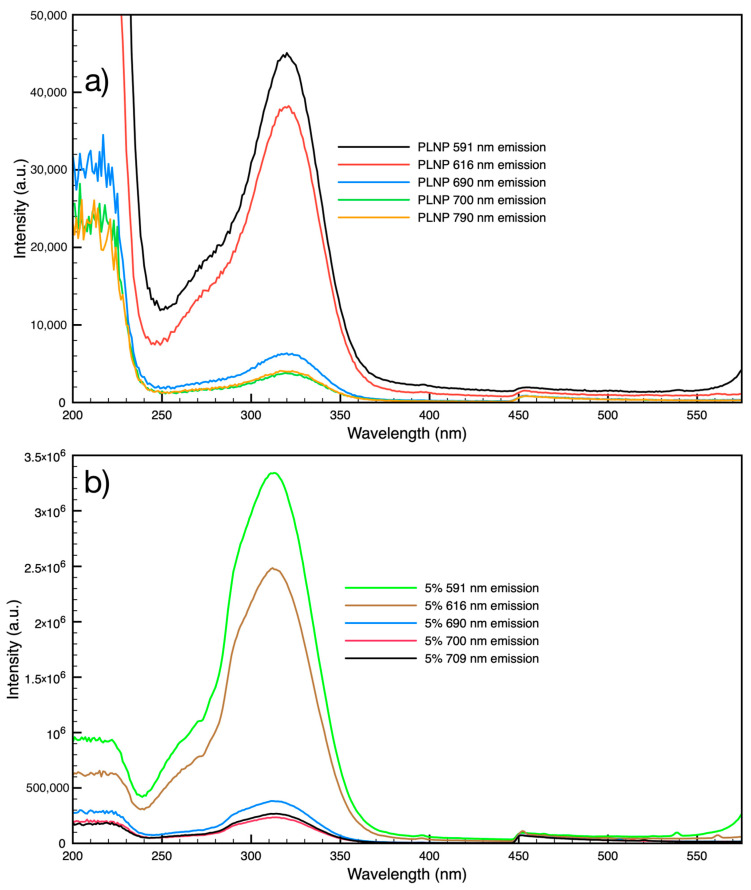
Excitation spectra for (**a**) PLNP powder sample emission peaks and (**b**) 5% PLNP-loaded filament emission peaks.

The PLNPs exhibited luminescence not only in the visible range but also in the near-infrared (NIR) region while under ultraviolet excitation (256 nm, 302 nm, and 325 nm) and short-lived persistent luminescence up to two minutes. The emission spectra of as-obtained PLNP powder and PLNP-loaded filaments show the typical visible emissions for Eu^3+^ with prominent peaks at 590 nm and 616 nm, corresponding to the ^5^D_0_ → ^7^F_1_ and ^5^D_0_ → ^7^F_2_ transitions for europium (Figure 1) [42]. Additional emission peaks are also seen in both the nanoparticles and PLNP-loaded filaments at 536 nm corresponding to the ^5^D_1_ → ^7^F_1_ transition, 555 nm corresponding to the ^5^D_1_ → ^7^F_2_ transition, and a manifold at 690 nm, 700 nm, 709 nm corresponding to the ^5^D_0_ → ^7^F_4_ transition. The PLNPs did show some weak but broad emission in the 330–450 nm range as well (see Figure 1 inset). The excitation spectra for the PLNPs (Figure 2a) exhibit a broad absorption band between 200 and 250 nm, another broad band with a peak around 320 nm, and another distinct peak at 397 nm. The slight bump at 450 nm is due to the long-pass filter used in the experimental setup. The excitation spectra for PLNP-loaded filaments (Figure 2b) showed similar features with a broad band from 200 to 230 nm, a prominent band with a peak at 313 nm, and a small peak at 397 nm.

**Figure 3:**
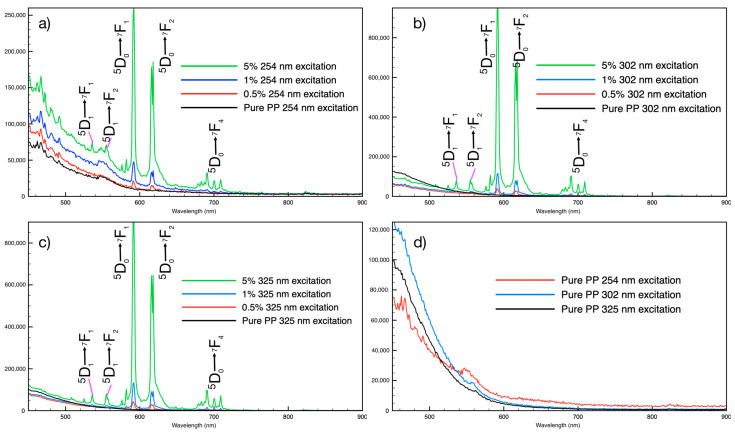
Emission spectra of PLNPs in extruded polypropylene filaments at different loading percentages under (**a**) 254 nm excitation, (**b**) 302 nm excitation, and (**c**) 325 nm excitation. (**d**) The emission of pure PP filaments under UV excitation.

**Figure 4:**
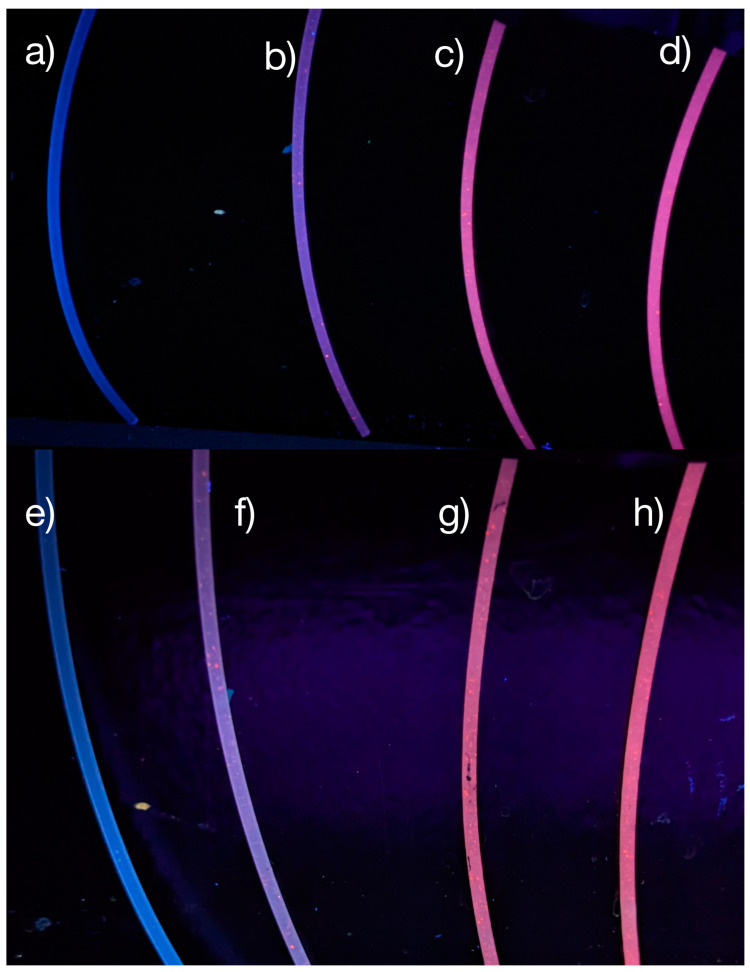
Filaments of various PLNP loading percentage under 302 nm excitation: (**a**) pure PP, (**b**) PP with 0.5% PLNP, (**c**) PP with 1% PLNP, and (**d**) PP with 5% PLNP initially, and (**e**) pure PP, (**f**) PP with 0.5% PLNP, (**g**) PP with 1% PLNP, and (**h**) PP with 5% PLNP after 24 h of continuous UV exposure.

While the optical properties of the PLNPs themselves are important to characterize and optimize for any given application, any differences in these properties once the PLNPs are embedded within the filament should also be examined. Putting the extruded filaments under the same ultraviolet excitation, the emission spectra revealed that there are no shifts to the NP emission peaks due to the filament environment (Figure 3). Unsurprisingly, the intensity of the emission spectra from the NPs within the filaments was less than for a pure PLNP powder sample. This is to be expected, as the PLNPs will spread out within the filament at the loading ratios used here; whereas, for a powder sample, the NP packing will be as high as possible, such that there are more emitting sites per surface area than in the filament samples. It is worth noting that even at the lowest loading percentage tested, 0.5% by weight, the visible emission is easily detected by spectrometer and by eye (Figure 4). Additionally, the filaments did not show any noticeable degradation or change in optical properties after prolonged exposure to UV light. The pure PP filaments exhibited some blueish-green emission, denoted by the broad emission around 450–500 nm in Figure 3d, as well as a small bump between 540 and 550 nm. These spectral features can also be seen in the spectra of the PLNP-loaded filaments. All three excitation wavelengths tested here resulted in emission from the PLNPs in the filaments.

### 3.2. PLNPs in Filaments

**Figure 5:**
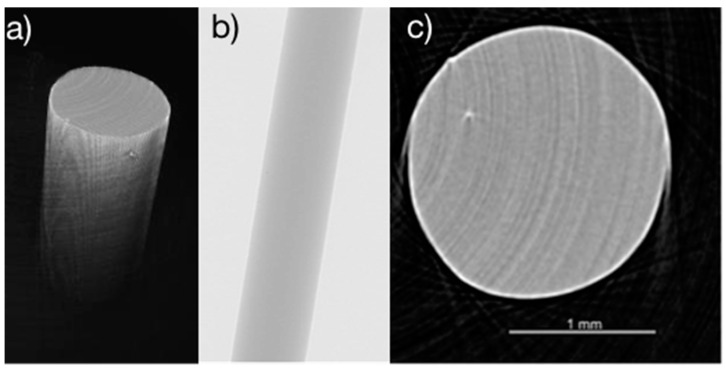
Optical microscopy views of pure polypropylene (PP) filament (**a**) at an angle; (**b**) from the side view; and (**c**) from the cross sectional view.

**Figure 6:**
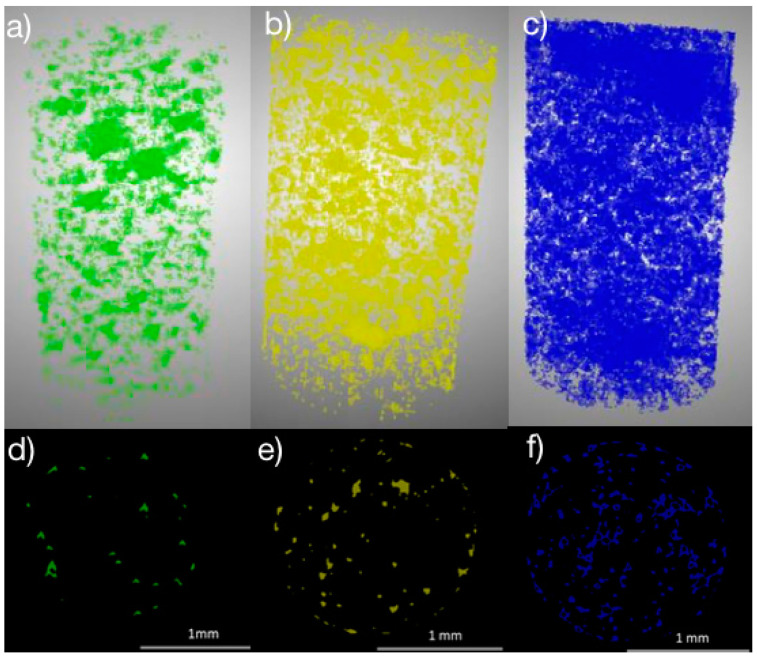
3D volumetric distribution of PLNPs in filaments at (**a**) 0.5%, (**b**) 1%, and (**c**) 5% loading by weight; and cross-sectional view of PLNP distribution in filaments at (**d**) 0.5%, (**e**) 1%, and (**f**) 5% loading by weight.

The distribution of nanoparticles when incorporated into filaments or textiles can have a dramatic effect on the overall mechanical and thermal properties of the luminescent filament [28,43]. For the pure polypropylene (PP) filaments, shown under an optical microscope in Figure 5, there were no visible particles or inhomogeneities from the extrusion process, indicating a complete melt during extrusion. From the micro-CT scans (Figure 6), it can be seen that the PLNPs are well dispersed throughout the 3D volume and cross section of the filaments regardless of the loading percentage. As the loading percentage increases, the distribution of particles becomes more dense. Although the PLNPs are relatively uniformly distributed inside the filament, there is some clumping present. However, it is important to note that the PLNPs are not confined to either the shallow surface layer nor the core of the filament, indicating a true mixture of PLNPs and polypropylene during the melt extrusion process. These findings are in agreement with comparable studies by Shi et al. which investigated PLNPs in polyacrylonitrile [44] and by Silva et al. which incorporated multi-walled carbon nanotubes (MWCNTs) into polylactic acid (PLA) filaments through a similar melt extrusion process [45].

**Figure 7:**
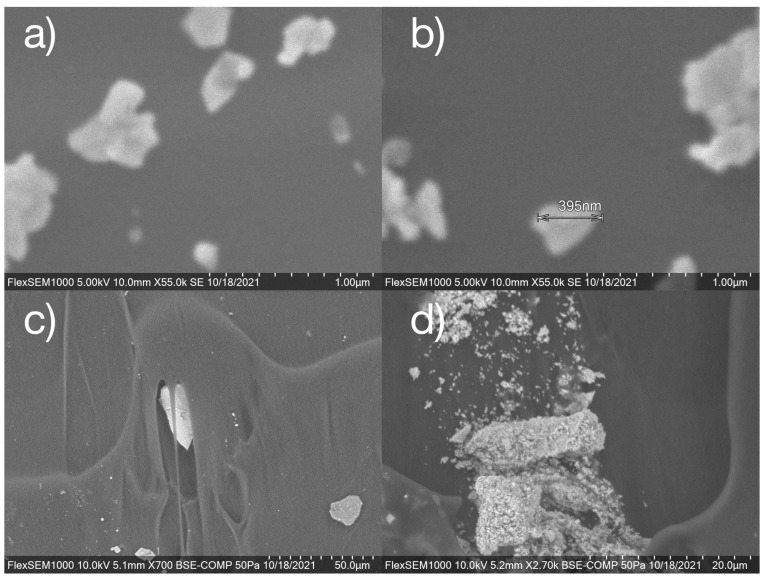
SEM images of (**a**,**b**) PLNPs in powder form and (**c**,**d**) PLNPs in PP filament.

Images of the PLNPs as a powder and incorporated into the filaments were also obtained by an environmental scanning electron microscope (Figure 7). It can be seen that the PLNPs range in individual size from sub-100 nanometer particles to 100’s of nanometers, although measuring exact sizes on the smaller particles was not possible given the resolution of the instrument. Individual PLNPs can be seen spread out through the cross section of the filaments (Figure 7c) while some clustering of PLNPs can be seen in certain parts of the filaments (Figure 7d).

### 3.3. Mechanical Analysis of Luminescent Filaments

Incorporation of nanoparticles can affect the mechanical properties of luminescent filaments in terms of the tensile properties. In this work, the breaking tenacity of filaments in terms of grams per denier (gpd), elongation at break (percentage), Young’s modulus (MPa), and secant modulus (MPa) were investigated to determine any effect of incorporating PLNPs. Linear density of the filament was measured from the mass of five different 185 mm lengths for each loading percentage. Table 1 exhibits the average denier of each sample. Five specimens of each type of filament loading (0, 0.5, 1, 5%) were evaluated.

**Figure 8:**
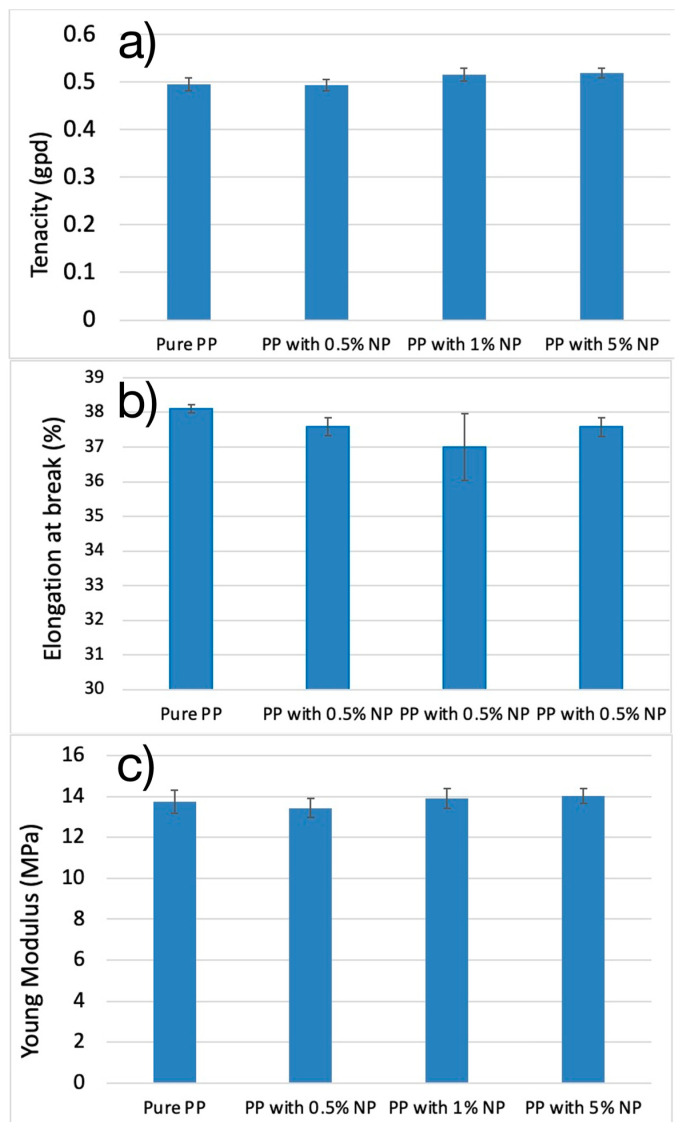
(**a**) Average tenacity at break for pure and PLNP-loaded filaments, (**b**) average elongation at break for pure and PLNP-loaded filaments, and (**c**) average Young Modulus for pure and PLNP-loaded filaments.

Figure 8 depicts the average tenacity, average elongation, and average Young’s modulus of pure PP and PP with varying NP percentages. Our results from the tensile properties analysis differ from some findings reported in the literature [35,39,46]. Similar studies found that the breaking strength and elongation at break of fibers reduce with the increased loading percentage of luminous NPs [34,47,48]. Conversely, our results indicated that the tenacity of the filaments increased slightly as the PLNP loading percentage also increased. Elongation percentage at break for all PLNP-loaded filaments was less than the pure PP filaments, but there was no clear trend over the loading range tested. Likewise, there was no statistically significant trend in the Young’s modulus for the loading range tested. The secant modulus (see Table 2), however, did decrease as the loading percentage increased. This indicates a loss of elasticity with more NP loading. It is worth noting that the filaments tested here were undrawn, and that drawn filaments may react differently under the mechanical testing conditions.

**Figure 9:**
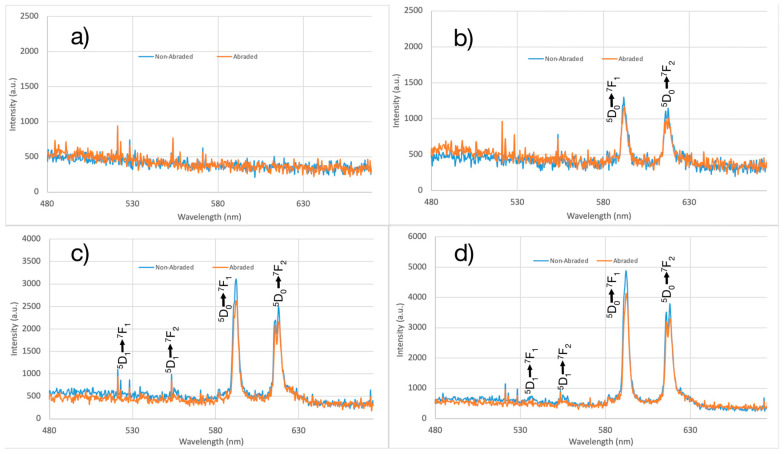
Emission spectra of abraded and non-abraded (**a**) pure PP sample filaments, (**b**) PP filaments with 0.5% PLNPs, (**c**) PP filaments with 1% PLNPs, and (**d**) PP filaments with 5% PLNPs.

During the use of luminescent filaments in fabric, normal wear and tear on the fabric can cause a degradation of the optical properties. Wear resistance was evaluated by comparing the emission spectra of abraded and non-abraded PLNP-loaded filaments. Filaments were abraded manually by using 80 grit sandpaper. To maintain the uniformity of the abrading action, ten equal alternating movements in opposite directions were performed, after which the samples were examined visually to ensure uniform abrasion. Figure 9a shows the visible emission spectra of pure PP for abraded and non-abraded samples which, unsurprisingly, did not show any luminescence in the infrared or near-infrared. Figure 9 also shows the emission spectra of abraded and non-abraded filaments with 0.5% PLNP, 1% PLNP, and 5% PLNP loading, respectively. Overall, the emission spectra of filaments showed that the abraded filaments also exhibit the same optical behavior as the non-abraded filaments, but due to the non-uniformity of the surface, they show lesser emission intensities. The findings from this study imply that when a fabric containing luminescent filaments is abraded, the optical behavior of the fabric will remain constant though the fabric may exhibit slightly lower emission intensity with a high degree of wear.

### 3.4. Persistent Luminescence

**Figure 10:**
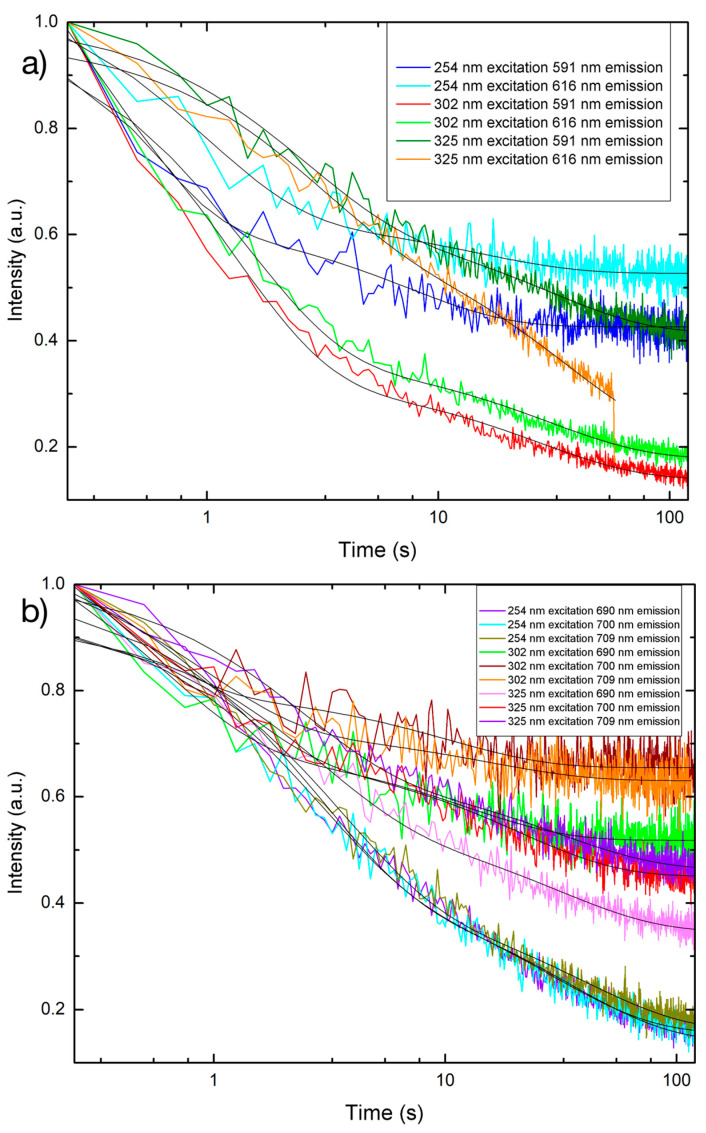
Semi-log plots of persistent luminescence decay curves for PLNPs in powder form for the (**a**) visible and (**b**) near-infrared regions. Black solid lines indicate the double-exponential decay fit.

The duration of luminescence can determine the most appropriate applications for PLNPs with a longer duration of persistence usually being more desirable for commercial applications. Once the PLNPs are charged, they can emit energy for a long period of time without any external energy source. While investigating the persistent luminescence time dependence for the PLNPs in powder form under different excitation wavelengths (Figure 10 and Table 3), it was found that the length of persistent luminescence for each emission wavelength can vary depending on the excitation. The visible persistent luminescence had a decay constant that ranged from 7 to 35 s and was still visible for up to 120 s before it became too weak to detect. The NIR persistent luminescence lasted for 30–60 s, depending on which specific wavelength, and had a decay constant ranging between 10 and 40 s. The shape of the decay curve also suggests that the luminescent intensity value decreased more sharply within the first 30 s, and afterwards that the intensity decreased at a slower rate, indicating a double-exponential decay and agreeing with results from the literature [49].

**Figure 11:**
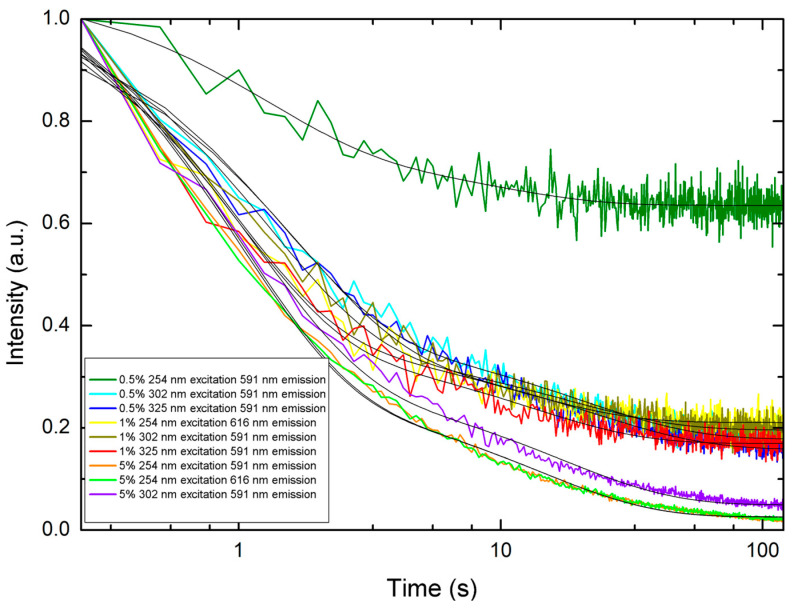
Semi-log plots of visible persistent luminescence decay curves for PLNP-loaded filaments. Black solid lines indicate the double-exponential decay fit.

**Figure 12:**
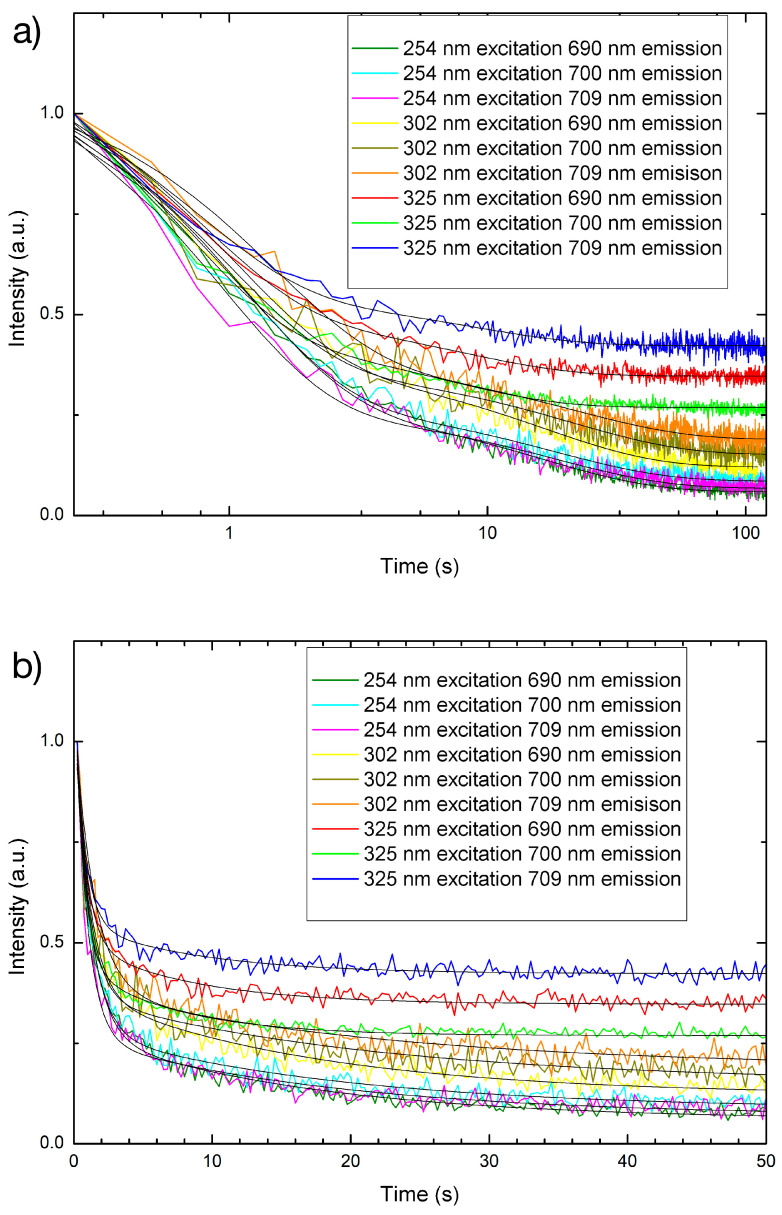
Near-infrared persistent luminescence decay curves for 5% PLNP-loaded filaments by weight over (**a**) 2 min on a semi-log plot and (**b**) enlarged view of first 50 s on a linear time scale. Black solid lines indicate the double-exponential decay fit.

The persistent luminescence from the PP filaments with PLNPs incorporated at different percentages (Figure 11 and Figure 12) shows that the emission is detectable even down to the lowest loading, 0.5% by weight. It can also be seen that the NIR persistent luminescence (Figure 12 and Table 4) from the filaments lasts on the order of 10–50 s before becoming too dim to be detected. This demonstrates that the filaments can be successfully engineered to exhibit persistent luminescence in the NIR. In general, the PLNP-loaded filaments demonstrated shorter decay constants than the PLNP in powder form (see Table 4). This indicates that there may either be some quenching effects from the polymeric matrix or other non-radiative effects occurring. There is one notable example that reverses this trend between the near-infrared decay constants for the PLNPs and the filaments: when under 302 nm excitation. Referring back to the PLNP emission in Figure 1, we can see that there are short wavelength emissions at 340 nm, 356 nm, 371 nm, and 382 nm when under 302 nm excitation. It is possible that some electrons excited to these energy levels may become trapped and contribute to the persistent luminescence phenomenon. When the PLNPs are embedded in the filaments and illuminated by 302 nm, however, some of the energy for these short wavelength emissions could be directly absorbed or resonantly transferred to the polymer instead of contributing to the persistent luminescence phenomenon. For the filaments as designed and tested here, exciting and charging under 254 nm yielded the longest NIR afterglow effect.

## 4. Conclusions

It has been demonstrated that persistent luminescent nanoparticles (PLNPs) can be easily incorporated into polypropylene (PP) filaments through a melt extrusion process. There were not any significant changes in the optical properties of the nanoparticles due to the polymeric environment of the filaments. The PLNP-loaded filaments could then be excited with UV light to exhibit visible and near-infrared persistent luminescence. Elongation at break and Young’s modulus did not change appreciably due to the incorporation of PLNPs into the filaments for loading percentages up to 5% by mass; however, the tenacity increased while the secant modulus decreased as the loading percentage increased, indicating a stiffening and slight loss of elasticity as the PLNP loading was increased. Micro-CT analysis shows a relatively uniform distribution of PLNPs throughout the filament volumes and cross sections regardless of loading percentage. However, this method of extrusion did result in some clumping of the PLNPs which could also be seen by the naked eye. Such clumping may be remedied by surface modification of PLNPs before incorporating into the filaments via extrusion. This work demonstrates the proof of principle for fabricating filaments with persistent luminescent properties in the visible and infrared regions which can be converted into fabrics and used for identification. Future work will explore converting the filaments into fabrics, lengthening the persistent luminescence decay time, as well as greater spectral specificity to only have persistent luminescence in the visible or infrared regions. Additionally, more in-depth characterization of the microstructure of the filaments is planned to better understand the effect of the PLNPs on the mechanical properties, but at the low concentrations tested here, the effect is likely minimal.

## 5. Patents

This work resulted in the following patent: George, Brian Robert, Brian Yust, and Abdur Rahaman Sk. “Persistent luminescent nanoparticle and articles comprising the same”. U.S. Patent 11,873,431, issued 16 January 2024.

## Figures and Tables

**Table 1:** Filament Size Determinations.

Sample PLNP Loading Percentage	Denier (g/9000 m)	Weight of 185 mm Length (g)	Diameter (mm)
0% (Pure PP)	11,950	0.245 ± 0.012	1.496 ± 0.0217
0.5%	11,960	0.246 ± 0.020	1.57 ± 0.022
1.0%	20,020	0.411 ± 0.033	1.88 ± 0.048
5.0%	20,050	0.412 ± 0.038	1.91 ± 0.050

**Table 2:** Secant Modulus for pure and PLNP-loaded filaments.

Sample PLNP Loading Percentage	Secant Modulus at 2% Strain (MPa)	Secant Modulus at 4% Strain (MPa)
0% (Pure PP)	3.47	4.25
0.5%	3.01	3.75
1.0%	1.79	2.83
5.0%	1.34	2.04

**Table 3:** Decay constants from double-exponential fitting of PLNP persistent luminescence decay curves.

Sample, Excitation Wavelength	Emission Wavelength	Decay Constant τ_1_ (s) ± Standard Error	Decay Constant τ_2_ (s) ± Standard Error
PLNP, 254 nm	591 nm	7.3994 ± 0.5766	0.3723 ± 0.0533
PLNP, 254 nm	616 nm	14.7525 ± 1.7455	0.9653 ± 0.1131
PLNP, 254 nm	690 nm	35.0124 ± 0.6946	2.6905 ± 0.0847
PLNP, 254 nm	700 nm	29.6682 ± 0.9390	2.6445 ± 0.1248
PLNP, 254 nm	709 nm	40.4209 ± 1.0176	3.3752 ± 0.1194
PLNP, 302 nm	591 nm	27.4936 ± 0.7038	1.2538 ± 0.0367
PLNP, 302 nm	616 nm	29.7887 ± 0.7798	1.4239 ± 0.0455
PLNP, 302 nm	690 nm	12.2215 ± 1.1750	0.5984 ± 0.1213
PLNP, 302 nm	700 nm	10.2954 ± 1.4042	0.3514 ± 0.1351
PLNP, 302 nm	709 nm	15.336 ± 2.9839	0.8897 ± 0.1938
PLNP, 325 nm	591 nm	30.1912 ± 1.5994	2.4726 ± 0.1629
PLNP, 325 nm	616 nm	35.9099 ± 0.5825	2.7200 ± 0.2876
PLNP, 325 nm	690 nm	30.9896 ± 0.14515	2.6832 ± 0.1719
PLNP, 325 nm	700 nm	20.7917 ± 0.8880	0.9743 ± 0.1017
PLNP, 325 nm	709 nm	31.1097 ± 1.4779	2.3018 ± 0.0195

**Table 4:** Decay constants from double-exponential fitting of PLNP-loaded filament persistent luminescence decay curves.

Sample, Excitation Wavelength	Emission Wavelength	Decay Constant τ_1_ (s) ± Standard Error	Decay Constant τ_2_ (s) ± Standard Error
5% loading, 254 nm	591 nm	14.0431 ± 0.2728	0.9938 ± 0.0196
5% loading, 254 nm	616 nm	13.9855 ± 0.2713	0.9700 ± 0.0197
5% loading, 254 nm	690 nm	16.2753 ± 0.4410	1.1097 ± 0.0306
5% loading, 254 nm	700 nm	18.1334 ± 0.6959	1.0957 ± 0.0425
5% loading, 254 nm	709 nm	18.2897 ± 0.5481	0.8909 ± 0.0288
5% loading, 302 nm	591 nm	17.0090 ± 0.3201	1.1343 ± 0.0254
5% loading, 302 nm	690 nm	16.5278 ± 0.6111	1.0273 ± 0.0473
5% loading, 302 nm	700 nm	21.1452 ± 0.8600	0.9468 ± 0.0545
5% loading, 302 nm	709 nm	21.6270 ± 1.2059	1.4183 ± 0.0773
5% loading, 325 nm	690 nm	7.9449 ± 0.5195	0.7432 ± 0.0425
5% loading, 325 nm	700 nm	6.9627 ± 0.3380	0.6313 ± 0.0276
5% loading, 325 nm	709 nm	8.6477 ± 0.7964	0.7321 ± 0.0581
1% loading, 254 nm	616 nm	11.4980 ± 0.7954	0.8799 ± 0.0619
1% loading, 302 nm	591 nm	17.4693 ± 0.9230	1.2430 ± 0.0585
1% loading, 325 nm	591 nm	12.9628 ± 0.5674	0.9274 ± 0.0423
0.5% loading, 254 nm	591 nm	8.7748 ± 2.0564	1.1866 ± 0.2717
0.5% loading, 302 nm	591 nm	20.6078 ± 0.6697	1.3994 ± 0.0543
0.5% loading, 325 nm	591 nm	22.7326 ± 0.7177	1.6070 ± 0.0543

## Data Availability

Data are contained within the article.
